# Distant organ metastasis patterns and prognosis of cervical adenocarcinoma: a population-based retrospective study

**DOI:** 10.3389/fmed.2024.1401700

**Published:** 2024-05-30

**Authors:** Suyu Li, Wuyuan Pan, Jianrong Song, Lan Zhen, Yusha Chen, Weijian Liu, Yulong Zhang, Lingsi Chen, Qiuyuan Huang, Shixiong Zheng, Xiangqin Zheng

**Affiliations:** ^1^Department of Radiation Oncology, Fujian Maternity and Child Health Hospital, College of Clinical Medical for Obstetrics & Gynecology and Pediatrics, Fujian Medical University, Fuzhou, China; ^2^Department of Gynecology, Fujian Maternity and Child Health Hospital, College of Clinical Medical for Obstetrics & Gynecology and Pediatrics, Fujian Medical University, Fuzhou, China; ^3^Department of Clinical Medicine, Xinxiang Medical University, Xinxiang, Henan, China; ^4^Fuzhou Second Hospital, National Clinical Research Center for Orthopedics, Sports Medicine & Rehabilitation, Fujian Provincial Clinical Medical Research Center for First Aid and Rehabilitation in Orthopaedic Trauma, Fuzhou, China

**Keywords:** cervical adenocarcinoma, SEER database, metastasis, prognosis, survival

## Abstract

**Background:**

Adenocarcinoma is a common histological subtype of cervical cancer, accounting for 10–15% of all cases. The prognosis of cervical adenocarcinoma with distant organ metastases remains unclear. Therefore, our study aimed to investigate the patterns and prognosis of distant organ metastasis in cervical adenocarcinoma.

**Methods:**

We obtained data from the Surveillance, Epidemiology, and End Results (SEER) database spanning from 2010 to 2019. Cox regression, Kaplan–Meier, and log-rank analyses were conducted.

**Results:**

We observed that adenocarcinoma (AC) of the cervix primarily metastasizes to single organs, with a rate of 73.3%. The lungs are the most common organs of metastasis, followed by the liver and bones. Patients with bone metastases have a median survival period of 12 months, which is slightly longer compared to metastasis in other organs. Distant organ metastasis, age, positive lymph nodes, higher AJCC stages, larger tumor diameter, and higher cell grades are related to poor prognosis (*p* < 0.001). Furthermore, we have observed that surgical intervention, radiotherapy, and chemotherapy can potentially provide benefits for patients with distant organ metastases.

**Conclusion:**

Metastasis is an independent prognostic factor for cervical adenocarcinoma patients. Surgery, radiotherapy, and chemotherapy can provide an overall survival advantage for patients with distant organ metastases.

## Introduction

Cervical cancer (CC) encompasses two main subtypes, Squamous Cell Carcinoma (SCC) and adenocarcinoma (AC), which are prevalent malignant gynecological tumors posing significant threats to women’s lives and health (1). While the incidence of squamous cell carcinoma has declined in developed countries, there has been a notable increase in both the relative and absolute rates of adenocarcinoma over the past few decades in certain European countries and developing nations. These rates have risen from 5% to 20–25% (2–5), with a tendency to affect younger individuals (6, 7).

Numerous studies have demonstrated that the 5-year overall survival rate for non-Squamous Cell Carcinoma (non-SCC) is 10 to 20% lower compared to SCC (8, 9). Additionally, adenocarcinoma carries a higher risk of recurrence. Among 24,562 patients with stage IB-IVB cervical cancer, AC exhibited a higher risk of mortality when compared to SCC. In early-stage disease (IB1-IIA), AC posed a mortality risk (hazard ratio = 1.39; 95% confidence interval, 1.23–1.56), while in late-stage disease (IIB-IVA), AC also had a mortality risk (hazard ratio = 1.21; 95% confidence interval, 1.10–1.32) (7). Furthermore, among 229 stage Ib AC patients, the rate of distant metastasis was significantly higher at 37% compared to 1,538 SCC cases at 21% (*p* < 0.01). In 636 patients with stage Ib-IIa large-volume cervical cancer, the recurrence rate for AC stood at 14.4%, which was significantly higher than that of SCC. AC exhibited a higher rate of recurrence in the blood/distant region (9/20; 45.0%), while SCC had a lower rate (8/36; 22.2%, *p* = 0.07) (10). Particularly noteworthy is the higher rate of distant metastasis observed in cervical cancer cases with high-risk factors (11), as defined by the “Sedlis Criteria,” which includes lymph node metastasis (LNM), surgical margin status, and parametrial involvement (12).

The heterogeneity of adenocarcinoma (AC) and its diverse histological subtypes have raised questions about its propensity for distant organ metastasis. According to the World Health Organization (WHO) in 2014, cervical adenocarcinoma is classified into three types based on traditional histopathological morphology and cytoplasmic characteristics: usual-type endocervical adenocarcinoma (EAC), mucinous adenocarcinoma, and special types of adenocarcinoma (13). However, only a limited number of studies have compared metastatic patterns and prognostic relationships, particularly in the context of distant organ metastases. Consequently, this paper focuses on AC with distant organ metastases using data from the surveillance epidemiology and end results (SEER) database. The objective is to analyze the prognostic differences among various sites of distant organ metastasis and evaluate the impact of surgical, radiotherapeutic, and chemotherapeutic interventions on metastatic adenocarcinoma.

The findings from this study may contribute to a better understanding of the metastatic behavior of AC and aid in the development of personalized treatment strategies for patients with metastatic adenocarcinoma. Additionally, the results could potentially inform clinical decision-making regarding the selection and sequencing of therapeutic interventions based on the specific sites of distant organ metastasis.

## Methods

### Study cohorts

Data for this retrospective cohort study were obtained from the SEER database, specifically the Incidence-SEER 18 Regs Research Data from November 2020 Sub (covering the period of 2010–2019 with variations). The SEER database is a comprehensive and valuable resource for cancer-related information, encompassing data from 18 United States registries that represent approximately 28% of the country’s population. With its detailed analysis data, it serves as an important tool for research in the field.

To access the necessary patient records, the SEER workshop has been granted authorization to retrieve information on surgeries, radiotherapy, chemotherapy, as well as other clinical and pathological details. The SEER*Stat software system, version 8.4.1, developed by the National Cancer Institute in Washington, United States, was utilized to extract the relevant data from the SEER database.

In order to ensure the validity of the study findings, strict inclusion and exclusion criteria were applied. Patients included in the study had to meet the following criteria: a confirmed diagnosis of malignant adenocarcinoma as their first primary tumor, with the diagnosis falling within the time frame of 2010–2019. Additionally, their survival months and cause of death had to be known. On the other hand, patients diagnosed with cervical intraepithelial neoplasia (CIN) or other histologic types of cervical cancer, including squamous cell carcinoma (SCC), adenosquamous carcinoma, neuroendocrine tumors, mesenchymal tumors, undifferentiated carcinoma, or mixed epithelial and mesenchymal tumors such as adenosarcoma, were excluded from the study. Cases diagnosed through autopsy or death certificate were also excluded. Furthermore, patients with missing data regarding age, survival time, and metastatic status were not considered for analysis.

As the SEER database is publicly accessible, Institutional Review Board (IRB) approval was deemed unnecessary for this study. However, to ensure compliance with ethical guidelines, the necessary authorizations and permissions were obtained from the SEER program to access and utilize the dataset.

### Data collection

**Figure 1:**
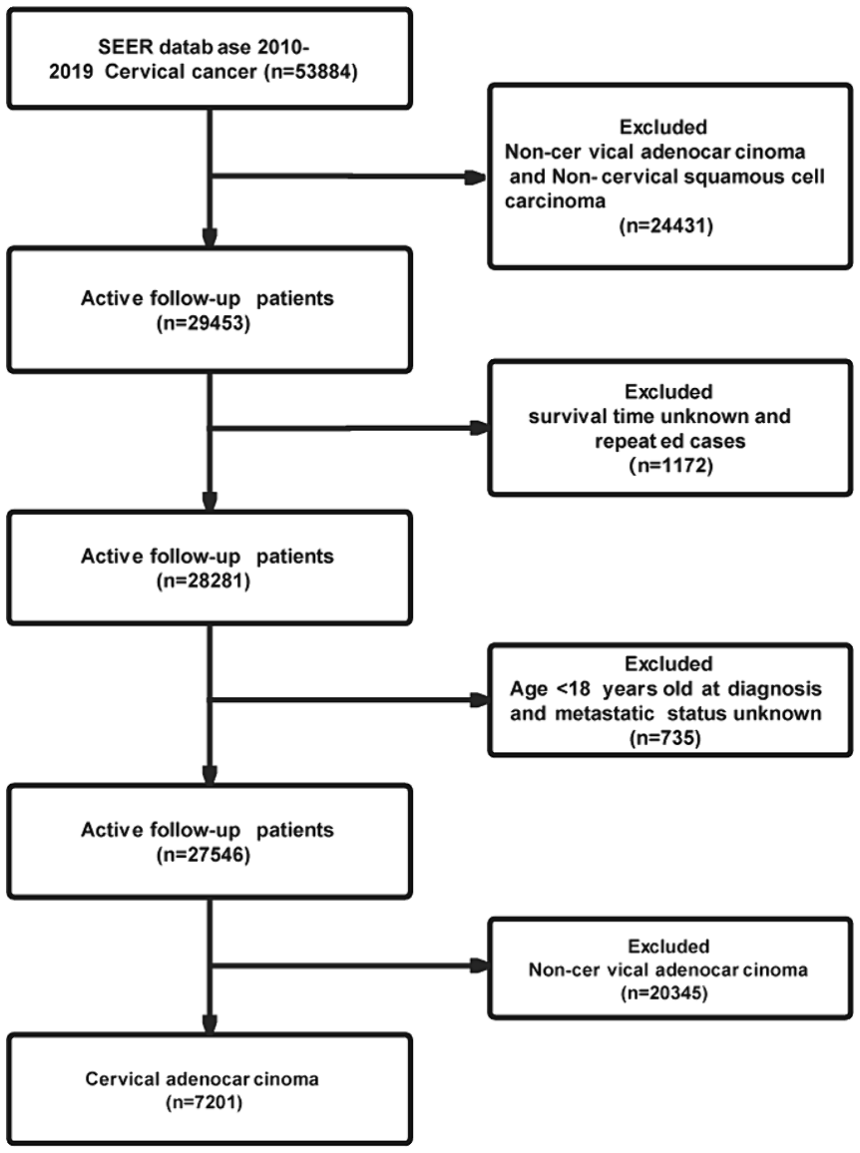
Flowchart of the cervical adenocarcinoma patient’s selection.

Data for the Cervical Adenocarcinoma study and relevant clinical information were retrieved from the SEER project. Patients diagnosed with first malignant primary site cervical cancer (Site record International Classification of Diseases for Oncology-3 (ICD-O-3)/WHO 2008: Cervix Uteri) between 2010 and 2019 were identified from the SEER database. Patients with International Classification of Diseases for Oncology (ICD-O-3) histology codes of 8,140, 8,141, 8,142, 8,144, 8,260, 8,262, 8,263, 8,310, 8,380, 8,441, 8,480, 8,490, 8,574 and 9,110 were included. Exclusion of cases with a pathological diagnosis was not cervical adenocarcinoma. Patients with unknown age (under 18), unknown follow-up time, or unclear metastatic status were excluded from the study. The flow chart depicting patient selection is shown in Figure 1.

The objective of this study is to conduct an in-depth analysis of the factors influencing Overall Survival in patients with Cervical Adenocarcinoma. In this study, a comprehensive approach was employed to investigate the associations among OS (overall survival) and various variables, including age, race, marital status, AJCC stage, grade, history, tumor size, positive lymph node, CSS(cause-specific death classification), cancer metastasis, and different treatments.

### Statistical analysis

To gain a deeper insight into the impact of these factors, a range of statistical methods were employed. For normally distributed measurement data, *t*-tests were utilized, while for skewed distribution data, the Wilcoxon rank-sum test was employed. Categorical data were analyzed using the χ2 test and the Kaplan–Meier and log-rank analyses.

All the analyses we test to explore differences and trends. For further study, subgroup analysis was applied to ascertain the extent of influence of age and history factors on Overall Survival of AC. By calculating odds ratios (ORs) with 95% confidence intervals (CIs), we objectively assessed the correlation between metastasis and overall survival.

The data analysis for this study was conducted using the R statistical software package (http://www.R-project.org, The R Foundation) and Free Statistics software version 1.8. The statistical methodology employed two-tailed tests to evaluate the significance of the results, with a *p*-value below 0.05 considered statistically significant.

### Group data

Age was initially considered a pivotal factor, and patients were classified into three groups: ≤18 & < 45, ≤45 & < 60, and ≥ 60. Tumor size was stratified into four categories: <2 cm, 2-4 cm, ≥4 cm, and unknown. Positive lymph nodes were classified into three groups: negative, positive, and unknown. History was classified into three groups: usual-type adenocarcinoma, mucinous adenocarcinoma (including 8,480-Mucinous adenocarcinoma, 8,481-Mucin-producing adenocarcinoma, 8,482-Mucin-producing adenocarcinoma, 8,144-Adenocarcinoma, intestinal type, and 8,490-Signet ring cell carcinoma), and other special types. We conducted a comprehensive analysis of these categorical variables and measurement data using statistical indicators such as frequency (percentages), mean ± standard deviation, median, extreme values, and interquartile range to provide a clear and concise description.

## Results

### Patient characteristics

A total of 53,884 patients diagnosed with cervical cancer between 2010 and 2019 were initially included based on the defined criteria. After excluding cases of non-cervical adenocarcinoma and non-cervical squamous cell carcinoma, a total of 29,453 patients remained in the study cohort. Subsequently, cases with unknown distant metastasis status, patients below 18 years of age, repeated cases, and non-cervical adenocarcinoma were further removed from the cohort analysis. Finally, the study cohort consisted of 7,201 cases, as shown in Figure 1. Table 1 presents detailed characteristics of the cohort, including patients with and without distant metastases in the Cervical Adenocarcinoma subgroup.

**Table 1:** Demographic and clinical characteristics for cervical adenocarcinoma patients diagnosed with and without distant metastasis (2010–2019).

Subject characteristics	Total *n* (%) *n* = 7,201	M1 (%) *n* = 6,732	M2 (%) *n* = 469	*p* value
Race				0.003
White	5,658 (78.6)	5,315 (79)	343 (73.1)	
Others	1,543 (21.4)	1,417 (21)	126 (26.9)	
Age (years)				< 0.001
18–45	2,794 (38.8)	2,739 (40.7)	55 (11.7)	
46–60	2,409 (33.5)	2,264 (33.6)	145 (30.9)	
>60	1,998 (27.7)	1,729 (25.7)	269 (57.4)	
Marital status				< 0.001
Married	3,531 (49.0)	3,352 (49.8)	179 (38.2)	
Others	3,670 (51.0)	3,380 (50.2)	290 (61.8)	
History				< 0.001
Usual-type adenocarcinoma	5,239 (72.8)	4,875 (72.4)	364 (77.6)	
Mucinous adenocarcinoma	704 (9.8)	682 (10.1)	22 (4.7)	
Other special type adenocarcinoma	1,258 (17.5)	1,175 (17.5)	83 (17.7)	
Tumor size				< 0.001
<2 cm	2,037 (28.3)	2,021 (30)	16 (3.4)	
2-4 cm	1,400 (19.4)	1,346 (20)	54 (11.5)	
>4 cm	1,710 (23.7)	1,543 (22.9)	167 (35.6)	
Unknown	2,054 (28.5)	1,822 (27.1)	232 (49.5)	
Positive lymph node				< 0.001
Negative	3,009 (41.8)	2,997 (44.5)	12 (2.6)	
Positive	538 (7.5)	516 (7.7)	22 (4.7)	
Unknown	3,654 (50.7)	3,219 (47.8)	435 (92.8)	
Stage				< 0.001
I	4,777 (66.3)	4,681 (69.5)	96 (20.5)	
II	692 (9.6)	692 (10.3)	0 (0)	
III	709 (9.8)	709 (10.5)	0 (0)	
IV	794 (11.0)	428 (6.4)	366 (78)	
Unknown	229 (3.2)	222 (3.3)	7 (1.5)	
Grade				< 0.001
I	1,892 (26.3)	1,856 (27.6)	36 (7.7)	
II	1,910 (26.5)	1,856 (27.6)	54 (11.5)	
III	1,135 (15.8)	1,009 (15)	126 (26.9)	
Unknown	2,264 (31.4)	2,011 (29.9)	253 (53.9)	
Surgery				< 0.001
Yes	4,920 (68.3)	4,871 (72.4)	49 (10.4)	
No	2,281 (31.7)	1,861 (27.6)	420 (89.6)	
Chemotherapy				< 0.001
Yes	2,873 (39.9)	2,578 (38.3)	295 (62.9)	
No	4,328 (60.1)	4,154 (61.7)	174 (37.1)	
Radiation				< 0.001
Yes	3,050 (42.4)	2,814 (41.8)	236 (50.3)	
No	4,151 (57.6)	3,918 (58.2)	233 (49.7)	
Bone				< 0.001
No	7,029 (97.6)	6,726 (99.9)	303 (64.6)	
Yes	151 (2.1)	0 (0)	151 (32.2)	
Unknown	21 (0.3)	6 (0.1)	15 (3.2)	
Brain				< 0.001
No	7,145 (99.2)	6,726 (99.9)	419 (89.3)	
Yes	33 (0.5)	0 (0)	33 (7)	
Unknown	23 (0.3)	6 (0.1)	17 (3.6)	
Liver				< 0.001
No	7,016 (97.4)	6,725 (99.9)	291 (62)	
Yes	165 (2.3)	0 (0)	165 (35.2)	
Unknown	20 (0.3)	7 (0.1)	13 (2.8)	
Pulmonary				< 0.001
No	6,895 (95.8)	6,718 (99.8)	177 (37.7)	
Yes	278 (3.9)	0 (0)	278 (59.3)	
Unknown	28 (0.4)	14 (0.2)	14 (3)	
Css				< 0.001
Alive or dead of other cause	5,698 (79.1)	5,570 (82.7)	128 (27.3)	
Dead attributable to this cancer	1,499 (20.8)	1,158 (17.2)	341 (72.7)	
Unknown	4 (0.1)	4 (0.1)	0 (0)	
Survival month, median (IQR)	33.0 (12.0, 69.0)	36.0 (14.0, 71.0)	7.0 (2.0, 15.0)	< 0.001

M1, with distant metastasis; M0, without distant metastasis; CSS, cause-specific death classification. The distant metastasis (M1) and no distant metastasis (M0) arms of the study were stratified based on the age of diagnosis, race, marital status, history, tumor size, positive lymph node, AJCC stage, treatments received (surgery, radiotherapy, chemotherapy). The two groups were subject to Pearson’s Chi-square statistical analysis.

The analysis revealed that the proportion of distant metastases was significantly higher in older women (≥61 years) compared to younger (18–45 years) and middle-aged (46–60 years) women (*p* < 0.001). Furthermore, significant differences were observed in other characteristics such as history, marital status, AJCC stage, grade, tumor size, positive lymph node involvement, different treatment modalities, cancer-specific survival (CSS), and overall survival (OS) between patients with and without distant metastasis (*p* < 0.001). Patients without distant metastases had a significantly higher rate of primary site surgery (72.4% vs. 10.4%, *p* < 0.001) compared to those with distant metastases. Conversely, patients with distant metastases had a significantly higher proportion of receiving primary site radiation therapy (50.3% vs. 41.8%, *p* < 0.001) and chemotherapy (62.9% vs. 38.3%, *p* < 0.001) compared to those without distant metastasis.

### Frequency of organ metastasis

The distribution characteristics of distal metastasis organs and their combined metastasis are shown in Table 2. Specifically, among the patients with single-organ metastasis, lung metastasis was the most common, accounting for 37.3% of the cases, followed by liver metastasis at 17.5%, and bone metastasis at 15.1%. Among all study subjects, approximately 26.7% of patients presented with multi-organ metastasis. Furthermore, the analysis showed that multi-organ metastasis involving the lung and liver predominantly accounted for 8.3% of all cases with multi-organ metastasis.

**Table 2:** Frequencies of different metastasis organs and combination metastasis (*n* = 469).

Metastatic site		Number	Percentage(%)
One organs	Bone	71	15.1
	Liver	82	17.5
	Pulmonary	175	37.3
	Brain	16	3.4
	Total	344	73.3
Two organs	Bone + Liver	15	3.2
	Bone + Pulmonary	29	6.2
	Bone + Brain	4	0.9
	Liver + Pulmonary	39	8.3
	Liver + Brain	1	0.2
	Pulmonary + Brain	5	1.1
	Total	93	19.8
Three organs	Bone + Liver + Pulmonary	25	5.3
	Bone + Liver + Brain	2	0.4
	Bone + Pulmonary + Brain	4	0.9
	Liver + Pulmonary + Brain	0	0.0
	Total	31	6.6
Four organs	All	1	0.2

### Univariate and multivariate analyses for cervical adenocarcinoma

This study utilized both univariate and multivariate Cox regression analyses to systematically assess potential prognostic factors in cervical adenocarcinoma patients with or without distant metastasis (Tables 3, 4). The results indicated that age at diagnosis, positive lymph node involvement, higher AJCC stages, larger tumor size, higher cell grade, and the presence of multi-organ metastasis were significantly associated with overall survival (OS) in both univariate and multivariate analysis.

**Table 3:** Univariable Cox regression analysis of overall survival in cervical adenocarcinoma patients with metastasis in SEER database (2010–2019).

Subject characteristics	Univariable	
	HR(95%CI)	*p*-value
Race		
White	ref	1.0
Others	1.49 (1.35, 1.65)	< 0.001
Age		
18–45	ref	1.0
46–60	2.64 (2.3, 3.04)	< 0.001
>60	6.74 (5.91, 7.69)	< 0.001
Marital status		
Married	ref	1.0
Others	1.54 (1.41, 1.69)	< 0.001
History		
Usual-type adenocarcinoma	ref	1.0
Mucinous adenocarcinoma	0.93 (0.8, 1.09)	0.383
Other special type adenocarcinoma	1.11 (0.99, 1.25)	0.064
Tumor size		
<2 cm	ref	1.0
2-4 cm	3.24 (2.66, 3.94)	< 0.001
>4 cm	7.43 (6.22, 8.88)	< 0.001
Unknown	5.81 (4.86, 6.94)	< 0.001
Positive lymph node		
Negative	ref	1.0
Positive	5.35 (4.45, 6.41)	< 0.001
Unknown	6.1 (5.34, 6.97)	< 0.001
Stage		
I	ref	1.0
II	2.52 (2.15, 2.95)	< 0.001
III	4.48 (3.94, 5.09)	< 0.001
IV	11.15 (9.93, 12.52)	< 0.001
Unknown	4.28 (3.44, 5.33)	< 0.001
Grade		
I	ref	1.0
II	1.52 (1.3, 1.77)	< 0.001
III	3.87 (3.34, 4.48)	< 0.001
Unknown	2.74 (2.38, 3.15)	< 0.001
Surgery		
Yes	ref	1.0
No	6.46 (5.86, 7.11)	< 0.001
Chemotherapy		
Yes	ref	1.0
No	0.39 (0.36, 0.43)	< 0.001
Radiation		
Yes	ref	1.0
No	0.51 (0.46, 0.56)	< 0.001
Bone		
No	ref	1.0
Yes	8.11 (6.74, 9.77)	< 0.001
Unknown	7.26 (4.67, 11.29)	< 0.001
Liver		
No	ref	1.0
Yes	10.32 (8.66, 12.3)	< 0.001
Unknown	15.52 (9.85, 24.46)	< 0.001
Pulmonary		
No	ref	1.0
Yes	9.32 (8.09, 10.74)	< 0.001
Unknown	9.74 (6.5, 14.58)	< 0.001
Brain		
No	ref	1.0
Yes	9.47 (6.42, 13.98)	< 0.001
Unknown	9.25 (6.01, 14.23)	< 0.001
Distant organ metastasis		
No transfer	ref	1.0
Bone	6.88 (5.23, 9.06)	< 0.001
Liver or pumonary	9.09 (7.83, 10.55)	< 0.001
Brain and multi-site metastasis	13.51 (11.18, 16.32)	< 0.001
Number of metastasis		
No transfer	ref	1.0
Single-site metastasis	8.49 (7.43, 9.69)	< 0.001
Multi-site metastasis	14.56 (11.95, 17.74)	< 0.001

HR, hazard ratio; CI, confidence interval; Ref, reference.

**Table 4:** Multivariable Cox regression analysis of overall survival in cervical adenocarcinoma patients with metastasis in SEER database (2010–2019).

Subject characteristics	Multivariable	
	HR (95%CI)	*p*-value
Metastasis		
No transfer	ref	1
Transfer	1.98 (1.69 ~ 2.31)	<0.001
Age		
18–45	ref	1
46–60	1.92 (1.66 ~ 2.22)	<0.001
>60	3.49 (3.04 ~ 4.01)	<0.001
Marital status		
Married	ref	1
Others	1.16 (1.06 ~ 1.28)	0.002
History		
Usual-type adenocarcinoma	ref	1
Mucinous adenocarcinoma	1.4 (1.2 ~ 1.64)	<0.001
Other special type adenocarcinoma	1.1 (0.98 ~ 1.24)	0.099
Tumer size		
<2 cm	ref	1
2-4 cm	1.87 (1.52 ~ 2.29)	<0.001
>4 cm	2.29 (1.88 ~ 2.78)	<0.001
Unknown	2.17 (1.79 ~ 2.62)	<0.001
Positive lymph node		
Negative	ref	1
Positive	2.3 (1.87 ~ 2.82)	<0.001
Unknown	2.01 (1.7 ~ 2.38)	<0.001
Stage		
I	ref	1
II	1.23 (1.04 ~ 1.46)	0.015
III	2.08 (1.8 ~ 2.42)	<0.001
IV	2.9 (2.49 ~ 3.38)	<0.001
Unknown	1.61 (1.28 ~ 2.02)	<0.001
Grade		
I	ref	1
II	1.23 (1.05 ~ 1.44)	0.009
III	1.82 (1.57 ~ 2.12)	<0.001
Unknown	1.27 (1.09 ~ 1.47)	0.002
Surgery		
Yes	ref	1
No	2.63 (2.29 ~ 3.03)	<0.001
Chemotherapy		
Yes	ref	1
No	1.42 (1.26 ~ 1.6)	<0.001
Radiation		
Yes	ref	1
No	1.31 (1.17 ~ 1.46)	<0.001

HR, hazard ratio; CI, confidence interval; Ref, reference.

Further results from the multivariate Cox regression analysis revealed several factors closely associated with a poor prognosis in cervical adenocarcinoma patients. These factors include the presence of distant metastasis (HR: 1.98, 95% CI: 1.69–2.31, *p* < 0.05), age over 60 (HR: 3.49, 95% CI: 1.88–2.78, *p* < 0.001), tumor diameter greater than 4 cm (HR: 2.29, 95% CI: 1.88–2.78, *p* < 0.001), higher tumor differentiation grade, and higher stage. Additionally, there was a 1.63-fold increased risk of not undergoing surgery (OR: 2.63, 95% CI: 2.29–3.03, *p* < 0.001).

**Figure 2:**
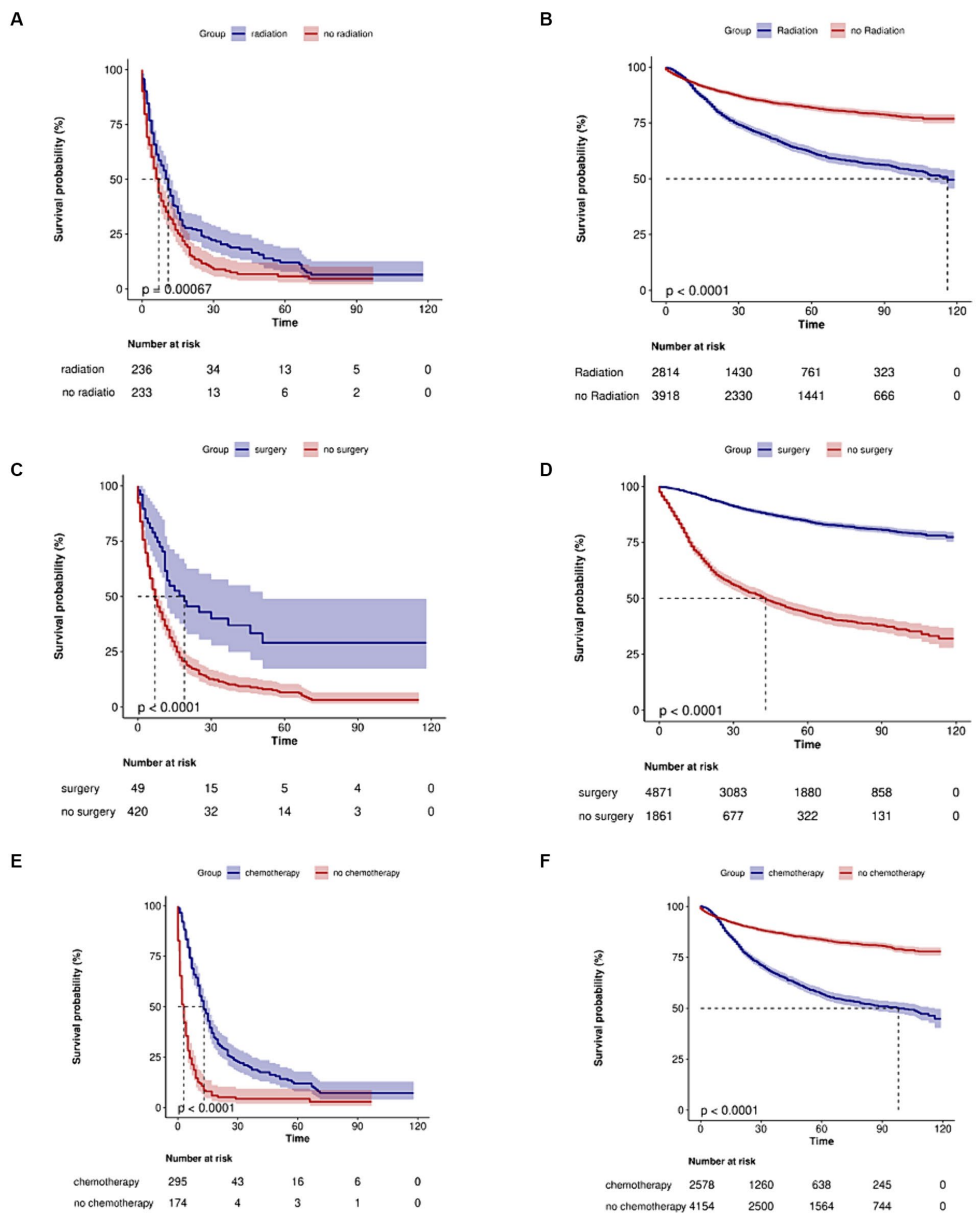
Kaplan–Meier curves of the overall survival in cervical adenocarcinoma with no metastasis or metastasis when stratified by treatment. **(A)** Radiotherapy in metastasis patients (*p* < 0.001). **(B)** Radiotherapy in no metastasis patients (*p* < 0.0001). **(C)** Surgery in metastasis patients (*p* < 0.0001). **(D)** Surgery in no metastasis patients (*p* < 0.0001). **(E)** Chemotherapy in metastasis patients (*p* < 0.0001). **(F)** Chemotherapy in no metastasis patients (*p* < 0.0001).

The effects of treatment on cervical adenocarcinoma patients with or without metastasis are illustrated in Figure 2. In the group without distant metastasis, the prognosis after surgery is notably better compared to cases without surgery. Conversely, in the group with distant metastasis, the median overall survival (OS) is significantly reduced for those who did not receive radiotherapy and chemotherapy, and this difference in median OS holds statistical significance.

**Figure 3:**
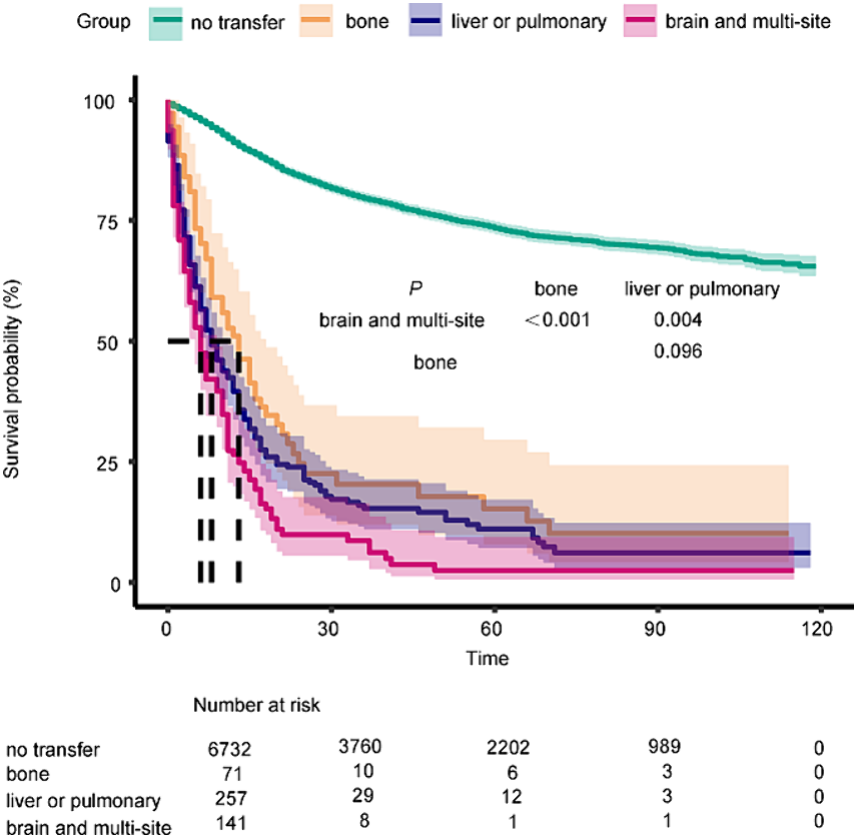
Kaplan–Meier curves of the overall survival in metastasis of different organs (no transfer, bone, liver or pulmonary, brain and multi-organ) (*p* < 0.0001).

Furthermore, the median survival time differs significantly among different types of distant metastasis, including bone metastasis, liver or pulmonary metastasis, and brain and other metastasis (*p* < 0.0001). In the group with distant metastasis, patients with bone metastasis have a significantly longer median survival period compared to patients with other types of distant metastasis, and this difference holds statistical significance (*p* < 0.0001) (Figure 3).

### Subgroup analyses

**Figure 4:**
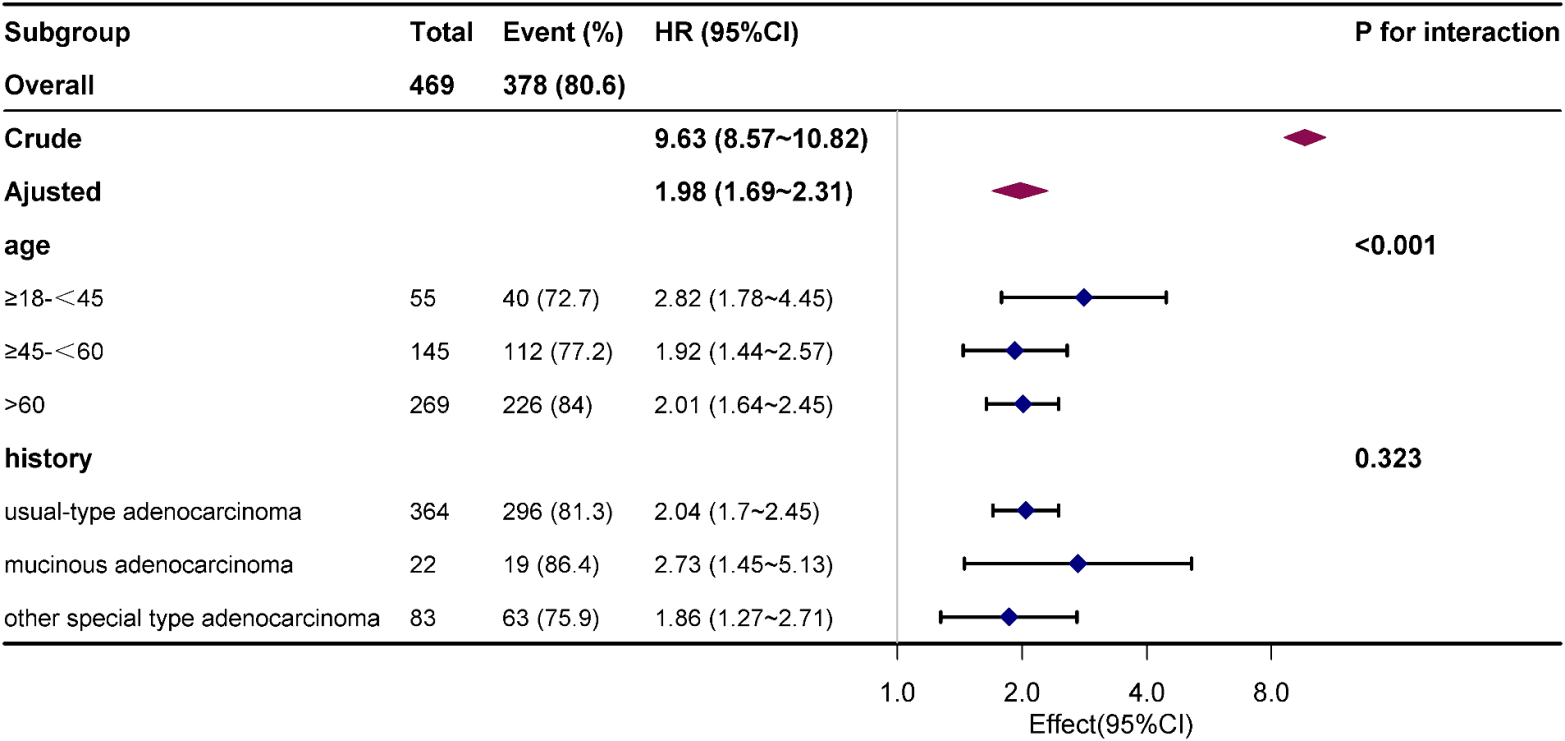
The effective size of age, history, surgery, radiotherapy and chemotherapy on overall survival in each group.

Combining the key conclusions mentioned above, the study results reveal a close relationship between age and post-metastasis survival rates. Particularly among patients aged ≤18 to 45, there is also a high risk of death (OR: 2.83, 95% CI: 1.79–4.46, *p* < 0.001). Patients with mucinous adenocarcinoma may have a higher susceptibility to metastasis (OR: 2.56, 95% CI: 1.37–4.77, *p* = 0.334), but further validation of this association is required (see Figure 4).

## Discussion

Among the three different types of adenocarcinoma, usual-type endocervical adenocarcinoma (EAC) is the most prevalent, accounting for 72.4% of cases with distant organ metastasis. Our study findings indicate that several factors are associated with an increased risk of distant organ metastasis in cervical adenocarcinoma. These factors include older age, larger tumor diameter, advanced clinical stage, higher histological grade, and the presence of lymph node metastasis (N1) at the time of diagnosis. Additionally, higher rates of distant metastasis were observed among individuals of white ethnicity and those who were unmarried. Consistent with findings in other solid tumors, our univariate and multivariate analyses identified several independent prognostic factors, including advanced age, higher T, N, M, and AJCC stages, larger tumor diameter, and higher cell grade (14–16). Furthermore, the occurrence of lung, liver, bone, and brain metastasis was found to reduce overall survival (OS) in patients. Multivariate Cox regression analysis confirmed that the presence of distant metastasis (HR: 1.98, 95% CI: 1.69 ~ 2.31, *p* < 0.05) negatively correlated with OS in patients with cervical cancer. Conversely, surgery, chemotherapy, or radiotherapy were shown to have a beneficial effect on patient prognosis. Taken together, these factors significantly impact the prognosis of cervical adenocarcinoma and should be carefully considered during treatment planning and patient management.

It is true that the metastasis pattern of adenocarcinoma (AC) differs from other pathological types of cervical cancer. Previous studies have indicated that lung and bone metastases are the most common organs of metastasis in cervical squamous cell carcinoma (17). On the other hand, for neuroendocrine carcinoma of the cervix (NECC), the liver and lungs are the most frequent organs of distant metastasis. NECC patients also tend to have a higher prevalence of multi-organ metastases, accounting for 44.4% of cases (18). In the case of adenocarcinoma, our study findings align with previous retrospective studies. We observed that adenocarcinoma more frequently exhibits single-organ metastases (73.3%). The most common organ of metastasis was the lungs (37.3%), followed by the liver (17.5%) and bones (15.1%). It is worth noting that multiple-organ metastases were observed in 26.7% of patients, with lung and liver metastases being the most common combination, followed by lung and bone metastases. Additionally, around half of the patients with brain metastases had concomitant metastases in other organs. The higher incidence of lung metastasis compared to brain metastasis may be attributed to the lung’s filtration of circulating cancer cells, while the brain’s distinct anatomical barrier and immune environment hinder cancer cell colonization (19). Brain metastasis is typically observed in advanced stages of cancer, after cancer cells have already disseminated to other organs. These distinct metastasis patterns highlight the importance of accurate radiographic assessment and a comprehensive evaluation of potential multi-organ metastasis in patients with adenocarcinoma.

The study shows that patients with isolated organ of bone metastasis have a median survival period of 12 months, which is significantly longer than patients with brain and & or those with multi-organ metastasis. This difference is statistically significant (*p* < 0.0001). The duration of survival observed in this study is slightly longer than the reported median survival of 7 months by Duangmani (20), possibly due to the separate analysis conducted in our study for isolated bone metastasis and multi-organ metastasis. Various treatment methods, including conventional radiotherapy, stereotactic body radiotherapy (21), and Magnetic Resonance-Guided Focused Ultrasound Surgery (22), are effective in achieving tumor control and biological ablation for the treatment of bone metastasis. Additionally, the use of osteoclast inhibitors can help reduce the risk of bone fractures (23). Surgical treatment is an option for patients requiring spinal decompression and open reduction and internal fixation (24). When multiple organ metastases are present, intravenous cisplatin combined with paclitaxel or carboplatin and paclitaxel chemotherapy is commonly used. However, survival after bone metastasis was longer in the patients who received radiotherapy (±chemotherapy) than in the patients who received chemotherapy alone as a salvage therapy (12 vs. 7 months; *p* = 0.01) (25). Our study supports the potential benefits of radiotherapy and chemotherapy in the treatment of metastatic patients. Primary site surgery which can help reduce tumor burden continues to be an important treatment option for eligible patients with advanced cervical cancer. It has been shown to improve survival rates in both metastatic and non-metastatic groups. Additionally, the latest NCCN guidelines have included targeted therapy and immunotherapy as treatment options for recurrent and metastatic cervical cancer (26). The addition of bevacizumab to chemotherapy significantly improved overall survival compared to chemotherapy alone, with a hazard ratio of 0.77 [95% CI 0.62–0.95], indicating a reduced risk of death, and a longer survival observed in patients without previous pelvic radiotherapy (27). Pembrolizumab exhibited substantial advantages in both progression-free survival and overall survival for patients receiving chemotherapy with persistent, recurrent, or metastatic cervical cancer. The study revealed a median progression-free survival of 10.4 months with pembrolizumab compared to 8.2 months with placebo, along with a higher overall survival rate of 53.0% at 24 months versus 41.7% in the placebo group (28).

Particularly noteworthy our subgroup analysis also indicates that young cervical cancer patients aged 18–45 years also have a high risk of death after distant metastasis (OR: 2.83, 95% CI: 1.79–4.46, *p* < 0.001). Some high-risk populations, such as those engaging in early sexual activity, having multiple sexual partners, smoking, having compromised immune function, and a history of sexually transmitted diseases, may be infected with multiple high-risk HPV types. This can progress to cervical cancer in the early stages and result in distant metastasis. Adisak also reported a statistically significant difference in overall survival between younger cervical cancer patients with bone metastases and older patients, with the median overall survival being shorter in the younger group (21 months, 95% CI 19.93–22.06; 34 months, 95% CI 23.27–44.72, *p* = 0.021) (29). Hence, for the purpose of enhancing both survival rates and quality of life, it is crucial not to underestimate the significance of comprehensive radiographic assessments in diagnosing cervical cancer among young patients. Young patients who can tolerate the associated side effects may derive potential advantages from more intensive and innovative multimodal treatment approaches.

Meanwhile, the study underscores the link between adenocarcinoma type and the risk of metastasis. The IECC consensus categorizes cervical adenocarcinoma into HPVA and NHPVA subtypes. NHPVA cervical adenocarcinoma has a worse prognosis compared to HPVA (30). The majority of mucinous adenocarcinomas belong to the non-HPV-related type, such as gastric-type adenocarcinoma (GAS) of the uterine cervix (31). Additionally, a retrospective study was published, which included 352 patients with usual-type endocervical adenocarcinoma (EAC) in stages I to IV (32). They classified these tumors using the Silva classification system: Pattern A (well-demarcated glands), B (early destructive stromal invasion arising from well-demarcated glands), and C (diffuse destructive invasion and more extensive lymphovascular space invasion). The findings demonstrated that Silva Type C tumors were associated with an increased risk of metastasis, recurrence, and mortality. Subsequent data consistently reinforces this association (33, 34). Consequently, the NCCN guidelines propose the utilization of the Silva classification system in combination with or integration into the FIGO/AJCC standards to facilitate clinical decision-making for identifying high-risk populations susceptible to metastasis (35).

Peritoneal and cutaneous metastases from cervical cancer are relatively rare, with retrospective studies indicating a frequency of approximately 1% for peritoneal metastasis and 0.1–2% for cutaneous metastasis (36, 37). Cutaneous metastasis appears to be more common in cervical adenocarcinoma compared to squamous cell carcinoma (36). These cases are typically reported in isolation. Various factors contribute to the risk of peritoneal spread, including the stage of the disease, histological type, and surgical technique. During minimally invasive surgery for cervical cancer, cancer cell spillage can occur due to several variables, including tumor exposure, the use of a uterine manipulator, and direct handling of the uterine cervix (38). For example, one report documented peritoneal metastasis in a cervical adenocarcinoma patient 16 months after undergoing laparoscopic surgery (39). The authors also compiled 13 cases of recurrence observed after laparoscopic surgery in cervical cancer patients. Consequently, ongoing research is concentrating on minimizing the risk of cell spillage during minimally invasive radical hysterectomy, with efforts directed towards optimizing the learning curve for laparoscopic surgery. However, Bogani et al. (40) found that, compared to minimally invasive surgery, open radical hysterectomy does not increase the incidence of 90-day surgery-related morbidity. Meanwhile, there are also numerous studies exploring improved minimally invasive surgical techniques, among which the modified laparoscopic radical hysterectomy (MLRH) approach has shown a survival advantage in patients with stage 1B1 and mid-third cervical invasion (41). Additionally, in another case of late-stage recurrent cervical cancer with peritoneal metastasis, 32 years after tumor resection, enhanced CT imaging revealed a large metastatic lesion in the right subphrenic space (42). Peritoneal lymphatic stomata, which link the peritoneal cavity to the lymphatic system, may contribute to the spread of the disease (43). Further research should focus on elucidating the mechanisms of metastasis in cervical adenocarcinoma and identifying biological markers for early detection of metastasis, as well as exploring novel diagnostic and therapeutic approaches to improve cervical cancer management.

Our study has several limitations that should be acknowledged. Firstly, the SEER database is a retrospective database. This introduces potential confounding biases as accurate staging is crucial for effective analysis. Moreover, there is a lack of clear information regarding HPV and Silva subtyping, as well as unclear data on the number and size of recurrent lesions, further complicating the assessment of treatment efficacy. Secondly, the database records do not include important indicators such as surgical methods, details of radiotherapy and chemotherapy cycles and doses, information on targeted therapies, immunotherapies, and patient complications. The absence of this crucial information limits our ability to comprehensively evaluate treatment outcomes and potential factors affecting prognosis. Thirdly, there is a lack of reporting on rare sites of metastasis, such as peritoneal metastasis and cutaneous metastasis, which require further attention. Therefore, it is necessary to consider these limitations when interpreting our study results. Further research is needed to address these knowledge gaps.

## Conclusion

Our findings indicate that the predominant pattern of metastasis in AC involves single organs, with lung metastasis being the most frequent, followed by liver and bone metastases. The median survival for patients with bone metastases was 12 months, slightly longer than for other organs of metastasis. We have also identified several independent prognostic factors for AC patients, which include the presence of metastasis, age, positive lymph node, higher AJCC stages, larger tumor size, and higher cell grade. Furthermore, our results demonstrate that surgery, radiation, and chemotherapy offer potential benefits for patients with distant organ metastases.

## Data availability statement

The original contributions presented in the study are included in the article/supplementary material, further inquiries can be directed to the corresponding authors.

## Ethics statement

Ethical approval was not required for the studies involving humans because the study utilized the public SEER database and was exempt from ethical review. The studies were conducted in accordance with the local legislation and institutional requirements. Written informed consent for participation was not required from the participants or the participants’ legal guardians/next of kin in accordance with the national legislation and institutional requirements because the study utilized the public SEER database and was exempt from informed consent.

## Author contributions

SL: Formal analysis, Methodology, Project administration, Writing – original draft. WP: Data curation, Formal analysis, Writing – original draft. JS: Investigation, Methodology, Supervision, Writing – review & editing. LZ: Resources, Writing – review & editing. YC: Resources, Software, Writing – review & editing. WL: Data curation, Writing – review & editing. YZ: Project administration, Visualization, Writing – review & editing. LC: Software, Supervision, Writing – review & editing. QH: Data curation, Formal analysis, Writing – review & editing. SZ: Methodology, Writing – review & editing. XZ: Funding acquisition, Investigation, Writing – review & editing.
